# Prevalence of scabies and impetigo in school-age children in Timor-Leste

**DOI:** 10.1186/s13071-021-04645-1

**Published:** 2021-03-15

**Authors:** Alexander Matthews, Brandon Le, Salvador Amaral, Paul Arkell, Merita Monteiro, Naomi Clarke, Terlinda Barros, Joaquim de Jesus Mendonça, Sonia Maria Exposto Gusmão, Leonia Maria dos Reis Seixas, João Henrique Araújo da Piedade, Daniel Engelman, Andrew C. Steer, Nicholas S. S. Fancourt, Jennifer Yan, John Kaldor, Joshua R. Francis, Susana Vaz Nery

**Affiliations:** 1 Royal Darwin Hospital, Darwin, Australia; 2 The Kirby Institute, University of New South Wales, Sydney, Australia; 3 Menzies School of Health Research, Charles Darwin University, Darwin, Australia; 4 Timor-Leste Ministry of Health, Dili, Timor-Leste; 5 Hospital Nacional Guido Valadares, Dili, Timor-Leste; 6 Tropical Diseases, Murdoch Children’s Research Institute, Melbourne, Australia

**Keywords:** Scabies, Impetigo, Children, Timor-Leste

## Abstract

**Background:**

Scabies and impetigo are endemic in many tropical, low- and middle-income countries. Mass drug administration (MDA) with ivermectin has emerged as a control strategy for these conditions. In 2019, Timor-Leste Ministry of Health planned to implement MDA including ivermectin for the control of lymphatic filariasis, so we undertook a baseline assessment of scabies and impetigo to better understand local epidemiology and contribute to future surveys assessing the impact of MDA.

**Methods:**

A cross-sectional school survey was conducted in April–May 2019 at six primary schools in a semi-urban (Dili) and two rural (Ermera and Manufahi) settings. Children under 19 years of age present at school on survey days were eligible to participate, of whom we enrolled 1183. Trained health workers interviewed and examined 1043 participants to clinically diagnose scabies using the 2020 International Alliance for the Control of Scabies (IACS) diagnostic criteria, as well as impetigo. Prevalence was adjusted for age and sex. Mixed-effects logistic regression models were used to analyse odds of scabies and impetigo infection. All models accounted for clustering at the school level through the use of random effect terms. Population attributable risk of scabies as a cause of impetigo was also estimated.

**Results:**

The overall weighted prevalence of scabies was 30.6%. Children in rural Manufahi were more likely to have scabies than those in semi-urban Dili (53.6% *vs* 28.2%, adjusted odds ratio [AOR] 3.5). Most cases of scabies were mild (3 to 10 lesions), and lesions were usually distributed on more than one body region. Scabies prevalence was lower among 10 to 14 year olds compared to 5 to 9 year olds. Overall weighted prevalence of impetigo was 11.3%. Relative to Dili, children in rural Ermera and Manufahi were twice as likely to have impetigo. Impetigo was twice as common in children with scabies than in those without, corresponding to an attributable risk of scabies as a cause of impetigo of 22.7%.

**Conclusions:**

Scabies and impetigo prevalence in Timor-Leste is among the highest reported globally, particularly in rural areas. Scabies infestation was strongly associated with impetigo. Comprehensive control strategies are urgently needed in Timor-Leste.
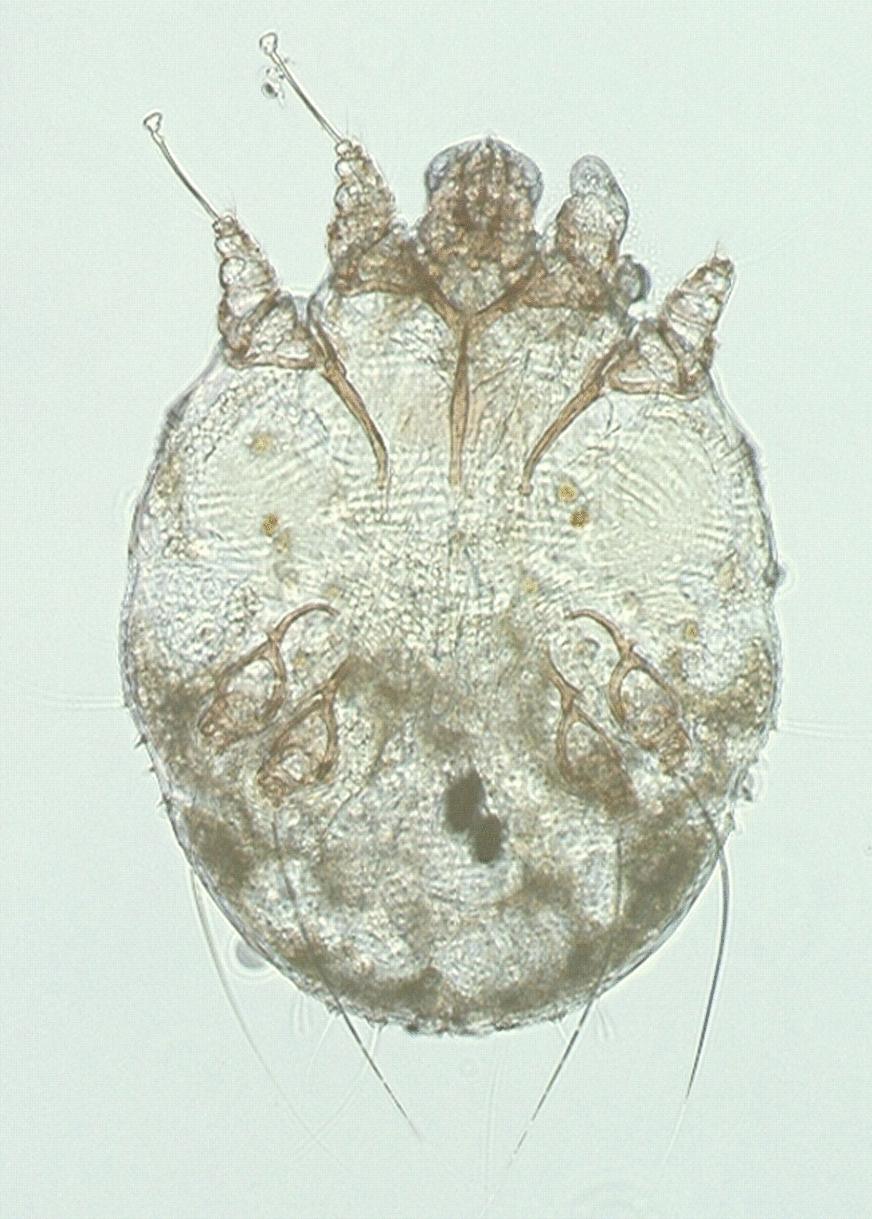

## Background

Scabies is a highly contagious skin condition caused by the mite *Sarcoptes scabiei* var. *hominis* [1]. In 2017, over 175 million people were estimated to be infested with scabies worldwide [2]. Scabies is recognised as one of the neglected tropical diseases, conditions for which effective interventions are available but that continue to cause significant morbidity in many resource-poor communities of tropical and sub-tropical countries [3]. Scabies infestation causes severe itch and skin lesions, including papules, nodules, burrows and vesicles. Transmission usually occurs through prolonged contact with infested skin [1]. Impetigo, a superficial bacterial skin infection, has been associated with scabies and can lead to more severe skin and soft tissue infections, invasive bacterial infections and post-streptococcal disease and is postulated as an aetiology of rheumatic fever and rheumatic heart disease (RHD) [3,[4]. Scabies and impetigo disproportionately affect infants, children, adolescents and, in some populations, the elderly (> 70 years of age) [2,[5].

Timor-Leste is a Southeast Asian nation of 1.2 million people that regained independence in 1999. There are 13 municipalities set in a tropical climate with mountainous terrain. Dili, the capital city, has a population of over 220,000 residents, within the namesake municipality of Dili, which includes urban and semi-urban areas [6]. Three surveys [7–[9] have been published since 1970 relevant to the epidemiology of scabies and impetigo in Timor-Leste. The most recent survey reported a prevalence of scabies and impetigo of 22.4% (312/1396) and 9.7% (136/1396), respectively, among school children in Dili and the rural municipality of Ermera in 2016 [7].

In 2019, following a World Health Organization recommendation, Timor-Leste Ministry of Health added ivermectin to an existing annual mass drug administration (MDA) program to eliminate lymphatic filariasis that consisted of annual distribution of diethylcarbamazine citrate and albendazole. Ivermectin is a broad-spectrum oral neuroinhibitory that paralyses and kills many parasites, including scabies and microfilariae [10], and has been shown in clinical trials to be highly effective as MDA for reducing scabies and impetigo prevalence in several island populations [11–[13].

In this study we assessed the prevalence of scabies and impetigo among children and young people aged < 19 years recruited in semi-urban and rural primary schools to better understand the epidemiology of each skin condition in Timor-Leste, particularly outside urban settings. The survey aimed to provide a baseline prevalence of these conditions prior to MDA and was the first in the Asian region to make use of consensus diagnostic criteria for scabies published in 2020 by the International Alliance for the Control of Scabies (IACS) [14].

## Methods

### Study design

**Fig. 1:**
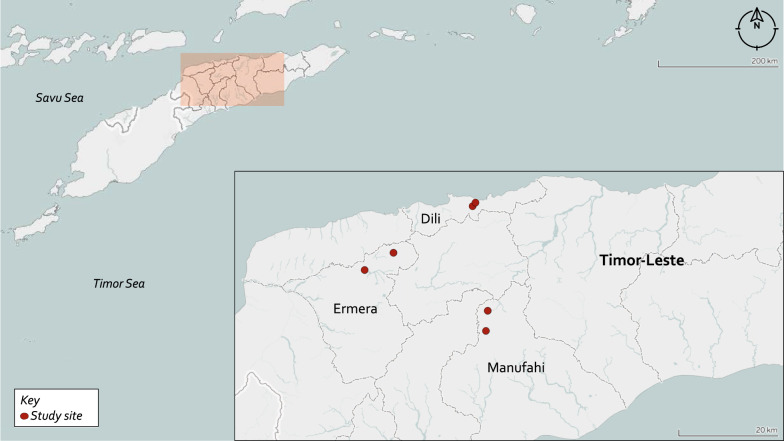
Study sites in Timor-Leste. The figure shows locations of the six primary schools, indicated by red pins, in the three municipalities included in the study. The inset is a magnification of the red box. The map was created using Mapbox in Tableau Public (2020)

In April and May 2019, we conducted a cross-sectional survey of children < 19 years of age in two primary schools in each of three municipalities (Fig. 1). One urban (Dili) and two rural (Ermera and Manufahi) municipalities were selected because they had previous prevalence data for comparison and provide socioeconomic and geographic diversity. Schools visited in Dili municipality were in the semi-urban administrative post of Cristo-Rei, outside the city. Primary schools that were accessible to the research team and had principals willing to engage in research were selected. A sample size of 801 was estimated to detect a prevalence of 22.4%, derived from the most recent survey [7], with 80% power, a confidence level of 95%, margin of error of 5% and design effect of 3.0 to adjust for cluster sampling. Epi Info software package (version 7.2) [15] was used to calculate sample size. School sizes were between 246 to 553 students. We aimed to recruit all children from each school to ensure the target sample size was met, given that there were concerns of lower school attendance and parent availability to provide consent close to Easter.

### Data collection procedures

Preparatory visits to participating schools were conducted to confirm that principals and teachers agreed that the survey could be conducted and to arrange for parents’ attendance. The survey took place over 2 consecutive days at each site. Parents were invited to be present on day 1 for explanation of the research, including questions and answers, and to provide verbal consent for their child(ren) to participate. Written consent was not required based on the preference expressed by school principals, clinical staff and other community leaders for this approach to limited skin examination, in keeping with previous local scabies research [7]. Trained health workers administered a standardized questionnaire to the children (or carers, for participants < 5 years old) and conducted a skin examination. Demographic data and assessment results were recorded on electronic tablets into a secure database (REDCap version 8.0 hosted at University of New South Wales) [16]. At one school, paper registration forms were also used and later entered into the database.

### Participants

All infants, children and adolescents < 19 years old present at schools on days of screening were eligible for enrolment in the study. This was inclusive of children and infants < 5 years old of families presenting at school. Families were not encouraged to bring younger children for screening but were informed on the study day that they were eligible to participate if present.

### Clinical diagnosis

Ten health workers (six Timor-Leste family medicine doctors, three visiting doctors and a visiting medical student) underwent a 1-day training program [17] in the diagnosis of scabies, impetigo and other skin conditions immediately prior to the study. The training was led by a doctor who practices in Northern Australia with multiple years’ experience in a scabies endemic population, with oversight from off-site paediatric infectious disease physicians. After training, all examiners passed a written assessment of 50 clinical photographs, meeting the minimum required competency of 80%. Clinical and suspected scabies were diagnosed using the 2020 IACS criteria [14]. In short, examination of exposed skin was conducted for scabies and impetigo lesions after removal of shoes in well-lit classrooms or outdoors. A diagnosis of scabies was only made if differential diagnoses were considered less likely. Severity of scabies was classified by number of lesions as very mild (1–2), mild (3–10), moderate (11–50) or severe (> 50). Affected body regions were classified into arms, face, legs and/or abdomen.

Impetigo was diagnosed if papular, pustular or ulcerative lesions with associated erythema, crusting, bullae or frank pus were observed [18]. Swabs of lesions were not collected given limitations of local laboratory facilities and resources. Severity of impetigo was classified by the number of lesions as very mild (1–5), mild (6–10), moderate (11–50) or severe (> 50).

Children diagnosed with scabies or impetigo were provided an information sheet in the national language (Tetum), describing the conditions and recommended treatments. Individual treatment for scabies was not provided as part of the study. Participants were advised of the planned Ministry of Health MDA for lymphatic filariasis, where all participants > 5 years old would be offered ivermectin (along with diethylcarbamazine citrate and albendazole), which is active against scabies. Children and infants < 5 years old not eligible for ivermectin were referred to health clinics for management. Individuals diagnosed with moderate-severe impetigo or other severe skin infections were referred to a local health clinic for management.

### Statistical analysis

Data were analysed using Stata version 14.2 (StataCorp, TX, USA). Unweighted and weighted prevalence of scabies and impetigo was calculated along with 95% confidence intervals (CIs). The weighted prevalence was adjusted for the population age and sex distribution in each municipality based on data retrieved from the 2015 Timor-Leste Population and Housing Census [6]. The population attributable risk (PAR) of scabies as a cause of impetigo was estimated using a standard formula [19].

Generalised linear mixed models were constructed with scabies and impetigo prevalence analysed as separate outcomes. Multicollinearity was investigated using variance inflation factors (VIFs). Predictor variables with a VIF > 5 were considered to indicate multicollinearity. Mixed-effects univariable and multivariable logistic regression analysis was undertaken with school entered as a random effect to account for clustering. The univariable regression was completed for each predictor variable with variables being retained for multivariable analysis if they had *p* < 0.2 on the Wald test. A forward variable selection approach was then used in a multivariable regression, with variables retained in the final model if they had *p* < 0.1 on the Wald test in the multivariable analysis. Adjusted odds ratios (AORs) were adjusted for age group and sex in all models. Cases with missing sex or age were excluded from regression models.

## Results

A total of 1043 children underwent skin examination. Half the participants were from semi-urban (Dili 47.7%) compared to rural sites (Ermera 34.8%; Manufahi 17.5%) (Additional file 1: Table S1). Where data were available, 53.8% were girls and 46.2% were boys. The median age was 10 years (interquartile range, 8–11 years, range, 8 months to 18 years). Sex and age were not recorded for 87 (8.3%) and 78 (7.5%) participants, respectively.

### Scabies

The weighted prevalence of scabies, adjusted for population sex and age, was 30.6% (95% CI 23.0–39.4). The unweighted prevalence was 33.4% (95% CI 30.6–36.3) (Table 1). Children aged 10 to 14 years were less likely to have scabies than 5 to 9 year olds (AOR 0.70, 95% CI 0.52–0.93, *p* = 0.01). There was no significant difference in prevalence across other age or sex groups. The odds of scabies infection were 3.5 times greater in Manufahi compared to Dili (53.6% [95% CI 46.3–60.7] *vs* 28.2% [95% CI 24.4–32.3], AOR 3.5, 95% CI 1.6–8.0, *p* = 0.02). There were no detectable significant differences in odds of scabies between Ermera and Dili (30.3% *vs* 28.2%, AOR 1.1, 95% CI 0.5–2.3, *p* = 0.9). Most diagnosed scabies cases were of mild (66.4%) or moderate (27.3%) severity. More cases met clinical scabies criteria (56.7%) than suspected scabies (43.3%). No cases of crusted scabies were identified.

**Table 1:** Scabies prevalence, severity, and diagnostic classification by municipality and age group

	Study sample, n	Scabies cases and unweighted prevalence, n (%)	AOR (95% CI)	Severity				Diagnostic classification		
				Very mild n (%)	Mild n (%)	Moderate n (%)	Severe n (%)	Clinical scabies B3 n (%)	Suspected scabies C1 n (%)	Suspected scabies C2 n (%)
Sex										
Male	442	156 (35.3)	Ref.	5 (3.2)	107 (68.6)	39 (25.0)	5 (3.2)	92 (59.0)	23 (14.7)	41 (26.3)
Female	514	167 (32.5)	0.9 (0.7–1.2)	5 (3.0)	111 (66.5)	48 (28.7)	3 (1.8)	91 (54.5)	32 (19.2)	44 (26.3)
Total n	956									
Age (years)										
0–4	25	13 (52.0)	1.1 (0.5–2.7)	0	7 (53.8)	6 (46.2)	0	9 (69.2)	2 (15.4)	2 (15.4)
5–9	445	170 (38.2)	Ref.	7 (4.1)	106 (46.4)	53 (31.2)	4 (2.4)	99 (58.2)	31 (18.2)	40 (23.5)
10–14	487	140 (28.7)	0.7* (0.5–0.9)	3 (2.1)	104 (74.3)	29 (20.7)	4 (2.9)	76 (54.3)	21 (15.0)	43 (30.7)
15–18	8	1 (12.5)	0.3 (0.0–2.8)	0	1 (100)	0	0	0	1 (100)	0
Total n	965									
Municipality										
Dili	497	140 (28.2)	Ref.	3 (2.1)	91 (65.0)	40 (28.6)	6 (4.3)	80 (57.1)	20 (14.3)	40 (28.6)
Ermera	363	110 (30.3)	1.1 (0.5–2.3)	5 (4.5)	74 (67.3)	31 (28.2)	0	58 (52.7)	21 (19.1)	31 (28.2)
Manufahi	183	98 (53.6)	3.5* (1.6–8.0)	5 (5.1)	66 (67.3)	24 (24.5)	3 (3.1)	60 (61.2)	15 (15.3)	23 (23.5)
Total	1043	348 (33.4)		13 (3.7)	231 (66.4)	95 (27.3)	9 (2.6)	198 (56.9)	56 (16.1)	94 (27.0)

NB. Discrepancy for sex, age, and overall totals exist because of missing demographic data. *AOR* adjusted odds ratio, *CI* confidence interval, *ref* reference category; * denotes significance at p < 0.05; B3: typical scabies lesions in a typical distribution and two history features consistent with clinical scabies; C1: typical scabies lesions in a typical distribution and one history feature

More than one-quarter of participants reported a household member (35.9%) or close contact (25.2%) with itch. Scabies was 18 times more likely in children who reported a household member or close contact with an itch (88.3%, AOR 18.2, 95% CI 10.7–31.2, *p* < 0.001) compared to those with no household member or close contact with an itch. Fewer participants reported a household member (27.6%) or close contact (15.1%) with a rash similar to the appearance of scabies. The odds of scabies was not significantly associated with having a positive contact history with a typical rash (70.8%, AOR 1.4, 95% CI 0.8–2.3, *p* = 0.14) compared to children without a positive contact history with a rash.

Most participants with scabies had lesions on more than one body region (64.7%), and lesions were most frequently located on the arms (54.2%) and legs (40.2%) (Table 2). There was no evidence of multicollinearity on all regression analyses performed.

**Table 2:** Frequency of scabies-infested body region by age group

	**Age (years)**					
	**0–2** ***n (%)***	**3–4** ***n (%)***	**5–9** ***n (%)***	**10–14** ***n (%)***	**15–18** ***n (%)***	**Total** ***n (%)***
Body region						
Arms	5 (45.5)	9 (45.0)	204 (53.1)	172 (56.6)	1 (50.0)	391 (54.2)
Legs	4 (36.4)	9 (45.0)	155 (40.4)	121 (39.8)	1 (50.0)	290 (40.2)
Face	2 (18.2)	2 (10.0)	19 (4.9)	10 (3.3)	0	33 (4.6)
Abdomen	0	0	6 (1.6)	1 (0.3)	0	7 (1.0)

Most participants (64.7%) had scabies lesions on > 1 body region. The 0 to 2 year old age group has been distinguished because typical distribution diagnostic criteria differ amongst infants ≤ 2 years old [14]

### Impetigo

The overall age- and sex-weighted prevalence of impetigo was 11.3% (95% CI 7.7–16.5). The unweighted prevalence was 12.5% (95% CI 10.6–14.6) (Table 3). There was no significant difference between sex and age groups. Relative to Dili, children were nearly twice as likely to have impetigo in the rural municipalities of Ermera (14.9% [95% CI 11.6–18.9] *vs* 8.7% [95% CI 6.5–11.5], AOR 1.9, 95% CI 1.2–3.0, *p* = 0.004) and Manufahi (18.0% [95% CI 13.1–24.3] *vs* 8.7%, AOR 2.2, 95% CI 1.3–3.7, *p* = 0.003). Of those with impetigo, 93.1% had very mild cases (< 5 lesions). Impetigo was two times more likely in children who reported a household member or close contact with an itch (56.8%, AOR 1.9, 95% CI 1.1–3.5, *p* = 0.03) compared to those with no household member or close contact with an itch. However, the odds of impetigo were not significantly associated with having a positive contact history with a typical rash (42.5%, AOR 0.9, 95% CI 0.5–1.6, *p* = 0.64).

**Table 3:** Impetigo prevalence and severity by municipality and age group

	**Study sample** **n**	**Impetigo cases and unweighted prevalence** ***n (%)***	**AOR** **(95% CI)**	**Severity**			
				**Very mild** ***n (%)***	**Mild** ***n (%)***	**Moderate** ***n (%)***	**Severe** ***n (%)***
Sex							
Male	442	65 (14.7)	Ref	59 (90.8)	5 (7.7)	1 (1.5)	0
Female	514	56 (10.9)	0.7 (0.5–1.1)	53 (94.6)	3 (5.4)	0	0
Total	956						
Age (years)							
0–4	25	7 (28.0)	2.0 (0.8–5.1)	6 (85.7)	1 (14.3)	0	0
5–9	445	58 (13.0)	Ref	54 (93.1)	3 (5.2)	1 (1.7)	0
10–14	487	56 (11.5)	0.9 (0.6–1.4)	52 (92.9)	4 (7.1)	0	0
15–18	8	1 (12.5)	0.8 (0.1–6.9)	1 (100)	0	0	0
Total	965						
Municipality							
Dili	497	43 (8.7)	Ref	40 (93.0)	3 (7.0)	0	0
Ermera	363	54 (14.9)	1.9* (1.2–3.0)	53 (98.1)	1 (1.9)	0	0
Manufahi	183	33 (18.0)	2.2* (1.3–3.7)	28 (84.8)	4 (12.1)	1 (3.0)	0
Total	1043	130 (12.5)		121 (93.1)	8 (6.2)	1 (0.8)	0

*AOR* adjusted odds ratio, *CI* confidence interval, *ref* reference category, *significance at *p* < 0.05

The prevalence of impetigo was twice as high in participants with scabies as in those without (63/348, 18.1% [95% CI 14.4–22.5] *vs* 67/695, 9.6% [95% CI 7.7–12.1], AOR 2.0, 95% CI 1.3–3.0, *p* = 0.001), corresponding to a PAR of scabies as a cause of impetigo of 22.7% (95% CI 10.9–32.9). There was no evidence of multicollinearity on all regression analyses performed.

## Discussion

This study identified a very high prevalence of scabies and impetigo among children in Timor-Leste. Weighted prevalence of scabies (30.6%) was higher than in previous large-scale surveys, including a 2016 school survey in Dili and Ermera (22.4%) [7] and a 2007 survey of schools, clinics and hospitals of Oe-Cusse, Bobonaro, Cova Lima and Dili (17.3%) [8]. Weighted prevalence of impetigo (11.3%) was also higher than in two surveys (9.7% [7]; 6.6% [8]).

Changes to our Dili sample population compared to the 2016 study may explain increases in the overall scabies prevalence. Scabies prevalence in Dili (28.2%) was markedly higher than recently reported (26/502, 5.2%) [7]; however, the prevalence in Ermera (30.3%) and Manufahi (53.6%) was comparable to previous findings in 2016 (286/894, 32.0%) [7] and 1970 (181/295, 61.3%) [9], respectively. In Dili, we surveyed two semi-urban primary schools outside the capital city while the only Dili school included in the 2016 study was in the urban city. This semi-urban population may in fact be more representative of rural settings, where our findings suggest children are at higher risk of scabies and impetigo than in urban settings. Migration during Holy Week and Easter celebrations may have led to overestimates in our Dili results. Families with scabies in Dili may be less inclined to travel, or rural residents attending the city may have increased communicable skin disease exposure or city-dwelling children visiting endemic rural regions may have returned infested with the mite.

Rural scabies prevalence was among the highest reported globally and comparable to rates reported in Fiji (32%) [20] and northern Australia (35%) [21], but fewer than in a small sample in Papua New Guinea (78%) [5, 22]. Children in Manufahi, the most remote municipality sampled, were more likely to have scabies and impetigo than those in in Dili. Participants in Manufahi and Ermera had nearly twice the odds of impetigo infection as children in Dili. Risk factors for scabies, including poverty, household crowding and poor access to healthcare, are more likely to predominate in less urban populations [19,[23]. Considering that over two-thirds of the national population reside in mountainous rural areas with limited access to health services similar to the situation in Ermera and Manufahi [6], our results suggest scabies may be a significant national child health problem. Moreover, availability of first-line scabicidal therapy, including topical permethrin and benzyl benzoate, is limited in Timor-Leste. Community management strategies are urgently required, with particular attention to at-risk rural populations. The impact of ivermectin, diethylcarbamazine citrate and albendazole MDA for lymphatic filariasis on scabies and impetigo has not been studied elsewhere. This survey will serve as baseline to assess change in prevalence of the skin conditions after the MDA program.

We identified a strong association between scabies infestation and impetigo infection. In our study, the PAR of impetigo because of scabies (22.7%) was less than values reported in the Pacific region, such as Solomon Islands (41.1%) [18]. Secondary bacterial infection of scabies lesions with group A streptococci has been implicated in post-infective glomerulonephritis, rheumatic fever and RHD [4]. While the attributable risk of scabies for these complications of impetigo is not currently known [10], Timor-Leste has among the highest globally reported rates of RHD [24]. Comprehensive control strategies for impetigo require national attention. Availability of impetigo treatment options, including intramuscular benzathine penicillin G and oral amoxycillin, are improving with limited stockouts nationally. Individual treatment is useful for reduction of impetigo in highly endemic regions [25] and should be available alongside investment in water, sanitation and hygiene programs, and community education.

Our study found 10–14-year-old adolescents were less likely to have scabies than 5–9-year-old children, in keeping with observed epidemiology globally [5]. Although our focus was school-aged children, scabies prevalence was very high in a small sample of 0 to 4 year olds (13/25, 52.0%). There may have been overestimation of prevalence in infants if families were more inclined to attend the survey with young children who they considered had skin conditions requiring medical attention.

We used a training and diagnostic method, the 2020 IACS criteria [14], novel to Timor-Leste but widely used in Pacific Island settings. In sites where this approach has been used, non-expert examiners, including clinical officers and nurses, who underwent brief training similar to our study diagnosed less scabies but with high specificity compared to consensus expert opinion [17, 26]. The approach is a valid tool for scabies diagnosis in population-level research, although additional studies are required to validate the criteria’s diagnostic accuracy [17].

There were limitations to our study. We were unable to include children absent from school on survey days, which may have underestimated the burden of disease and missed cases of crusted scabies, which frequently precludes school attendance [27]. Participant numbers were lower than the number of students enrolled because of low student attendance around the Easter holiday period. Not all registered children underwent skin examination. Competing school priorities for students and, on occasion, a high number of children relative to research staff meant participants were not always examined on the same day as registration, which led to missed examinations where children did not return to school the subsequent day. Examination was limited to visible skin which may have contributed to underdiagnosis of skin conditions and underestimates of severity, but this was most appropriate for privacy and cross-sectional study. Confirmation of clinical and suspected scabies diagnoses with direct observation of the mite or its products by dermoscopy, skin scrapings and microscopy was not used as this was a pragmatic field survey and these tools are usually not available in clinical practice.

## Conclusions

Scabies and impetigo prevalence are very high among children in Timor-Leste and among some of the highest estimates reported worldwide, particularly in rural and remote areas. Scabies infestation was strongly associated with impetigo, which may contribute to considerable infective and post-infective complications in this population. Community scabies and impetigo control strategies are urgently needed in Timor-Leste, as is assessment of the impact on scabies of the MDA campaign to eliminate lymphatic filariasis that included ivermectin.

## Supplementary Information

**Additional file 1: Table S1.** Participation data for six schools in three municipalities.

## Data Availability

The datasets analysed during the current study are available from the corresponding author on reasonable request.

## Abbreviations

AOR: Adjusted odds ratio
CI: Confidence interval
IACS: International Alliance for the Control of Scabies
MDA: Mass drug administration
PAR: Population attributable risk
RHD: Rheumatic heart disease
