# Supplementary material for: Prevalence of scabies and impetigo in school-age children in Timor-Leste
Source: Parasit Vectors. 2021 Mar 15;14:156. doi: 10.1186/s13071-021-04645-1 (PMC7962383; doi:10.1186/s13071-021-04645-1)
Supplement: Supplementary file 1 — Additional file 1: Table S1. Participation data for six schools in three municipalities. [file 13071_2021_4645_MOESM1_ESM.docx]

| **Additional file 1: Table S1.** Participation data for six schools in three municipalities. | | | | | | | | |
| --- | --- | --- | --- | --- | --- | --- | --- | --- |
|  | **Enrolled students n** | **Registered in study**  **n (% of Enrolled)** | **Examined**  **n (% of Registered)** | **Male**  **n (%)** | **Female**  **n (%)** | **Gender not reported**  **n (%)** | **Average age (yrs)** | **Age not reported**  **n (%)** |
| **Manufahi** | |  |  |  |  |  |  |  |
| 1 | 246 | 65 (26.4) | 58 (89.2) | 29 (50.0) | 28 (48.3) | 1 (1.7) | 8.9 | 1 (1.7) |
| 2 | 252 | 147 (58.3) | 125 (85.0) | 59 (47.2) | 59 (47.2) | 7 (5.6) | 8.0 | 3 (2.4) |
| **Ermera** | |  |  |  |  |  |  |  |
| 3 | 286 | 199 (34.7) | 192 (96.5) | 78 (40.6) | 104 (54.2) | 10 (5.2) | 9.8 | 8 (4.2) |
| 4 | 304 | 174 (57.2) | 171 (98.3) | 82 (48.0) | 78 (45.6) | 11 (6.4) | 9.1 | 11 (6.4) |
| **Dili** |  |  |  |  |  |  |  |  |
| 5 | 553 | 413 (74.7) | 340 (82.3) | 123 (36.2) | 167 (49.1) | 50 (14.7) | 10.0 | 48 (14.1) |
| 6 | 312 | 185 (59.3) | 157 (84.9) | 71 (45.2) | 78 (49.7) | 8 (5.1) | 9.4 | 7 (4.5) |
| **Total** | **1953** | **1183 (60.6)** | **1043 (88.2)** | **442 (42.4)** | **514 (49.3)** | **87 (8.3)** | **9.4** | **78 (7.5)** |

NB. Percentage of Examined participants is reported for Male, Female, Gender not reported, and Age not reported
